# Evaluation of a Silver-Embedded Ceramic Tablet as a Primary and Secondary Point-of-Use Water Purification Technology in Limpopo Province, S. Africa

**DOI:** 10.1371/journal.pone.0169502

**Published:** 2017-01-17

**Authors:** Beeta Ehdaie, Chloe T. Rento, Veronica Son, Sydney S. Turner, Amidou Samie, Rebecca A. Dillingham, James A. Smith

**Affiliations:** 1 Department of Civil and Environmental Engineering, Unviersity of Virginia, Charlottesville, Virginia, United States of America; 2 Department of Microbiology, University of Venda, Department of Chemistry, University of Venda, Thohoyandou, Limpopo Province, South Africa; 3 Department of Medicine, University of Virginia, Charlottesville, Virginia, United States of America; Purdue University, UNITED STATES

## Abstract

The World Health Organization (WHO) recognizes point-of-use water treatment (PoUWT) technologies as effective means to improve water quality. This paper investigates long-term performance and social acceptance of a novel PoUWT technology, a silver-infused ceramic tablet, in Limpopo Province, South Africa. When placed in a water storage container, the silver-embedded ceramic tablet releases silver ions into water, thereby disinfecting microbial pathogens and leaving the water safe for human consumption. As a result of its simplicity and efficiency, the silver-embedded ceramic tablet can serve as a stand-alone PoUWT method and as a secondary PoUWT to improve exisitng PoUWT methods, such as ceramic water filters. In this paper, three PoUWT interventions were conducted to evaluate the silver-embedded ceramic tablet: (1) the silver-embedded ceramic tablet as a stand-alone PoUWT method, (2) ceramic water filters stand-alone, and (3) a filter-tablet combination. The filter-tablet combination evaluates the silver-embedded ceramic tablet as a secondary PoUWT method when placed in the lower reservoir of the ceramic water filter system to provide residual disinfection post-filtration. Samples were collected from 79 households over one year and analyzed for turbidity, total silver levels and coliform bacteria. Results show that the silver-embedded ceramic tablet effectively reduced total coliform bacteria (TC) and *E*. *coli* when used as a stand-alone PoUWT method and when used in combination with ceramic water filters. The silver-embedded ceramic tablet’s performance as a stand-alone PoUWT method was comparable to current inexpensive, single-use PoUWT methods, demonstrating 100% and 75% median reduction in *E*. *coli* and TC, respectively, after two months of use. Overall, the the filter-tablet combination performed the best of the three interventions, providing a 100% average percent reduction in *E*. *coli* over one year. User surveys were also conducted and indicated that the silver-embedded ceramic tablet was simple to use and culturally appropriate. Also, silver levels in all treated water samples remained below 20 μg/L, significantly lower than the drinking water standard of 100 μg/L, making it safe for consumption. Long-term data demonstrates that the silver-embedded ceramic tablet has beneficial effects even after one year of use. This study demonstrates that the silver-embedded ceramic tablet can effectively improve water quality when used alone, or with ceramic water filters, to reduce rates of recontamination. Therefore, the tablet has the potential to provide a low-cost means to purify water in resource-limited settings.

## Introduction

Drinking water for approximately 1.8 billion people around the world is contaminated with fecal pathogenic microorganisms, and thus contributes to the 2 million deaths annually associated to diarrheal diseases [1]. Furthermore, water-related diseases can cause cognitive impairment and growth stunting [2] among children under the age of five. Lack of access to safe water is a significant barrier to improvement in human health and development of the global community. Households may have access to public taps monitored by the municipal government, however, they often face water shortages and impaired municipal water systems leading to poor drinking water quality [3]. Point-of-use water treatment (PoUWT) methods have shown great promise in improving water quality at the point of consumption. Currently, there are multiple promising PoUWTs, including ceramic water filters [4–6], chlorine-based methods [7,8], biosand filters [9,10] and flocculent/disinfecting powders [11]. Although proven effective in a laboratory setting, the performance of these PoUWT methods is dependent on external factors, such as user-compliance, ease of use of the technology, and existing poor sanitation and hygiene conditions, when used in households [12]. For example, chlorine-based methods usually alter the taste and odor of water, which can be unappealing to many end-users [7,8,11,13]. PoUWT methods such as flocculent/disinfecting powders have short lifespans or require multiple operating steps and supplies. Even with ceramic water filters, studies have shown recontamination of treated water can occur within a month of use due to poor sanitation conditions [14]. As a result, long-term performance of PoUWT methods can decline when applied in field settings [15,16].

The silver-embedded ceramic tablets described by Ehdaie et al [17] show potential in addressing some of the challenges seen with current PoUWT methods. The novel, low-cost method of embedding silver in ceramic media is placed in a water storage container, where it releases silver ions into water for microbiological disinfection. It provides residual disinfection at levels safe for consumption and can be reused for at least 6 months [17]. For the first time, this paper evaluates the silver-embedded ceramic tablets performance as a PoUWT method in a field setting. The silver-embedded ceramic tablet can be used as in two ways: as a stand-alone PoUWT method for individuals in need of a low-cost, reusable and portable PoUWT method and as a secondary PoUWT method to provide continual disinfection to reduce risks of recontamination. An example of a secondary PoUWT application would be with ceramic water filter systems. Ceramic water filter systems consist of a porous, pot-shaped ceramic filter inside a 20-L plastic, water storage container. When water is passed through the filter, particulates and microorganisms are physically removed and the treated water is then stored in the water storage container, referred to as the lower reservoir. As mentioned previously, the treated water is at risk of recontamination due to poor sanitation conditions. PoUWT methods, such as the silver-embedded ceramic tablet, have the potential to provide residual disinfection post-filtration by simply being placed in the lower reservoir.

To better understand the potential of the silver-embedded ceramic tablet as an effective PoUWT method, this paper compares three PoUWT interventions: (1) the silver-embedded ceramic tablet when used alone (SCT), (2) ceramic water filters when used alone (CWF), and (3) a filter-tablet combination, where the silver-embedded ceramic tablet is placed in the lower reservoir of the ceramic water filter system to provide continual disinfection (CWF+SCT). Each intervention method has its own advantages and disadvantages. Using the ceramic tablet as the primary PoUWT methods will microbiologically improve water quality, however not remove turbidity. The ceramic water filter removes pathogens and particulates but treated-water is at risk of recontamination. The filter-tablet combination removes microbial pathogens and turbidity while the silver-embedded ceramic tablet provides continual disinfection to reduce recontamination, but has yet to be field tested. This paper compares the technical performance and social acceptance of these three PoUWT methods among 79 households in Limpopo province, South Africa.

## Materials and Methods

### Ethics

The protocol for this study was approved by the University of Virginia Institutional Review Board for Social and Behavioral Sciences. With the assistance of interpreters, verbal consent was obtained from each participant prior to the beginning of the study. A written consent form was read to each participant and the interpreter recorded the respond of participants. Most participants were illiterate, therefore the verbal consent was used, and consent was recorded on a written consent form for this reason. This procedure was approved by the ethics committee. Participants were informed of all details of the study. For those using the silver-embedded ceramic tablet as the stand-alone PoUWT method, participants were informed that one of the ceramic tablets they were given was embedded with silver and the other was not but participants were not told which was which. Participants were instructed to use ceramic tablets and the corresponding containers only for storage of untreated water. Prior to consumption, they should treat their water using whatever water treatment methods they currently use, since the efficacy of the silver-embedded ceramic tablet is still being evaluated in this study. All participants were informed that silver is a disinfecting agent and, when used to treat water, would disinfect waterborne pathogens. All participants were provided with a silver-embedded ceramic tablet and ceramic water filter at the end of the study.

### Field site and enrollment eligibility

The field study was conducted in Limpopo Province, South Africa. Limpopo Province was selected as the location of the study because it is one of the least developed provinces in South Africa with the highest rates of HIV/AIDS and lowest rates of accessible drinking water (44%) [18]. It is home to the Water and Health in Limpopo (WHIL) program, an interdisciplinary collaboration between the University of Virginia and University of Venda. The WHIL project aims to bring safe drinking water to improve community health in the Limpopo Province.

A total of 79 households were randomly selected from two rural villages in Limpopo Province to participate in the study. To be eligible, participants had to be at least 18 years of age and without previous exposure to any of the PoUWT intervention technologies. Households who were eligible and interested were identified with assistance of the chief in the village. From this group of eligible participants, households were selected at random. Prior to being enlisted, participants were screened for previous knowledge or exposure to any of the intervention technologies in the study. If households had previously owned or extensively used one of the intervention technologies they were not eligible to participate.

### Intervention and design of study

**Fig 1:**
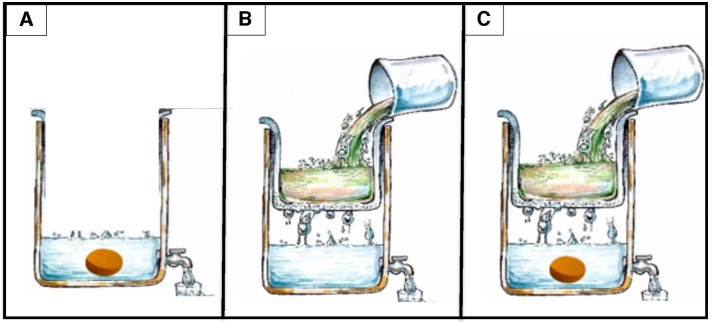
Ceramic-based point-of-use water purification methods. (A) Silver-embedded ceramic tablet in 20-L lower reservoir (SCT). (B) Ceramic water purification system: ceramic water filter and lower reservoir (CWT). (C) Ceramic water purification system with silver-embedded ceramic tablet in lower reservoir (CWF+SCT).

The performance of the silver-embedded ceramic tablet was evaluated as the stand-alone PoUWT method (Fig 1A) among 29 households. Each of these 29 households were provided two 20-L water storage containers, one with a silver-embedded ceramic tablet (Fig 1A) and another with a control ceramic tablet (without silver), referred to as the SCT-only group. The container with the control ceramic tablet served as a blind control. Participants were asked to use both containers equally to the best of their ability. Also, participants were instructed to only use containers for collection and storage of untreated water.

Fifty households were given a ceramic water filters. Half of the households were also given a silver-embedded ceramic tablet that was placed in the lower reservoir of the ceramic water purification systems. These were the filter-tablet combination households (CWF+SCT) (Fig 1). The ceramic water filter served as the primary PoUWT method by physically removing contaminants, while the silver-embedded ceramic tablet served as the secondary PoUWT. The remaining 25 households were given only the ceramic water filter system, without a silver-embedded ceramic tablet, and thus served as the control group (CWF-only).

Households were visited weekly for five consecutive weeks and again at weeks 37 and 52. Households were instructed not to clean the lower reservoirs and water storage containers in order to evaluate the continual disinfection capability of silver-embedded ceramic tablets. An outline of the field study is shown in S1 Fig. Only 30 of the 79 households were sampled at week 37 due to time constraints and afterwards they were given new silver-embedded ceramic tablets and removed from study. The remainder of the households were visited at week 52 for sample collection. Once the sample was collected, they were provided with a new silver-embedded ceramic tablet and removed from the study. The original ceramic tablets were transported back to the University of Virginia for further laboratory testing.

### Preparation of ceramic water filters and ceramic tablets

Ceramic water filters were provided by the University of Virginia non-profit organization PureMadi, and were manufactured at the PureMadi filter factory located in Limpopo Province, S. Africa. The factory is a collaborative project between WHIL, the local Mukondeni Women’s Pottery Cooperative and PureMadi. Ceramic water filters were made with local supplies: clay from a natural clay deposit, locally processed sawdust and water. Clay was processed at the factory using a hammer mill. Sawdust was passed through a 16-mesh sieve and water from a private borehole was used. 68 kg of clay and 8 kg of sawdust were mixed for 30 min then 28 L of water was added and mixed for another 30 min. The mixture was molded in to a pot shape using a filter press, and air-dried for three days. Filters were fired using a wood-fire kiln. To ensure filters are working properly, the flow rates of the filters were tested. Filters were soaked in a water bath and then filled to the rim. Water levels were measured after 1 h and flow rates confirmed to be between 1.5 L/h to 3 L/h. All working filters were painted with 0.3 g of colloidal silver and allowed to dry. The factory also provides the lower reservoir that is used with the ceramic water filters. Lower reservoirs were prepared by drilling a hole in a 20-L Evernu bucket to attach the spigot. The filter and lower reservoir are sold as a single unit as shown in 2 1.

Silver-embedded ceramic tablets were made in Charlottesville, Virginia in a pilot production facility. A dry clay-sawdust mix was prepared with 168.8 g of Redart clay and 18.8 g of sawdust, passed through 20-mesh sieve. Silver nitrate solution (27.2 g/L) was prepared using deionized water and 57.6 mL was added to the dry mix. The wet mixture was molded and pressed for 1 min at 6,895 kPa using a hydraulic manual press, and air-dried for 72 h at room temperature. A total of 54 silver-embedded ceramic tablets were prepared and fired in an electric kiln (Evenflow). Ceramic tablets were fired at 150°C/h until 600°C and then at 300°C/h until 900°C and held for 3 h. For households in the village of Ha-Mashamba, control tablets were also provided that were prepared using deionized water in place of silver nitrate solution. All participants were given, a 20-L plastic water storage container with a spigot with each ceramic tablet.

### Sample collection

Sterile Whirlpak stand up sample bags were used to collect and transport 500 mL of each sample. Samples were stored in coolers with ice during transportation from sample site to the laboratory and analyzed within 6 hours of collection. From both villages, samples were taken from the water source where participants filled their water storage containers. Among households with the ceramic water filter intervention, source water samples (influent) represented the untreated water, and were compared to samples collected directly from the spigot of ceramic water filters and filter-tablet combination (effluent).

Among households with the silver embedded ceramic tablet as their primary PoUWT method, the source water represent local water sources (public taps, boreholes, etc). It was compared to samples taken from the water storage container that had the ceramic tablet without silver (blind control) and samples collected from the water storage container containing the silver-embedded ceramic tablet. All samples were collected in duplicate. Source water samples were collected on a weekly basis for weeks 1–5. Control- and silver-ceramic tablet-treated samples were collected at weeks 1–5, 37 and 52.

The total number of houses visited each week varied due to availability of the residents. A few households were removed from the study because they relocated over the span of the study. The total number of houses visited and samples collected for each week are recorded in S1 and S2 Tables.

### Water-quality testing

Water samples were analyzed for total silver concentration, turbidity, total coliform bacteria (TC) and *Escherichia coli (E*. *coli)*. Total silver concentrations were measured using a PerkinElmer HGA 900 graphite furnace atomic absorption spectrometer (GFAA) with a silver cathode lamp. Prior to GFAA analysis, samples were acid digested using nitric acid for a final sample concentration of 1% HNO_3_. Samples were prepared in South Africa and transported to the University of Virginia for GFAA analysis.

Turbidity was measured using the Hach 2100AN Turbidimeter. Samples were poured in glass vials, provided by the test kit, and shaken thoroughly to ensure particles were uniformly distributed. Prior to readings, the outer surface of the glass vial was wiped with silicone oil to ensure no scratches or smudges interfered with the reading. Results were measured in units of nephelometric turbidity units (NTU) using standards provided by the kit. In between samples, glass vials were rinsed three times with deionized water.

Membrane filtration techniques were used to quantify TC and *E*. *coli* in water samples. TC and *E*. *coli* were used as indicators of fecal contamination because they are commonly found in mammalian feces. 100 mL of sample, or diluted sample, was passed through a sterile 0.45-μm Millipore membrane filter using a vacuum pump. The filter paper was placed in a sterile Millipore petri dish containing m-Coliblue24 broth growth media (Millipore) and incubated overnight at 37°C. After 24 hours the dishes were counted for TC colonies, indicated by a red dot, and *E*. *coli* colonies, indicated by a blue dot. Bacterial counts were used to calculated arithmetic mean and geometric mean of bacteria concentrations each week. During each membrane filtration analysis, a negative control sample was prepared, which was cooled boiled-water. Negative controls never had bacteria, as was expected, ensuring there was no contamination of samples or supplies.

### Laboratory analysis of ceramic tablets

Ceramic tablets were collected from houses visited during week 37 and reanalyzed for silver release in the laboratory at the University of Virginia. Ceramic tablets were placed in 10 L of tap water in a 20-L plastic container, and sampled after 24 h. Silver-embedded ceramic tablets were run in parallel with corresponding control ceramic tablets. Samples were analyzed for total silver concentration using the GFAA, as described previously.

### Social acceptance

Social acceptance was evaluated through collection of survey data. Participants were asked to complete entrance and exit surveys. Willingness to pay was evaluated through a series of questions in the exit survey using the binning method [19]. Interpreters were used to assist in language and literacy barriers. Entrance surveys were structured to collect demographic data and information on current water practices. Exit surveys were designed to collect information on performance and potential demand of the ceramic-based water purification technologies.

## Results

**Fig 2:**
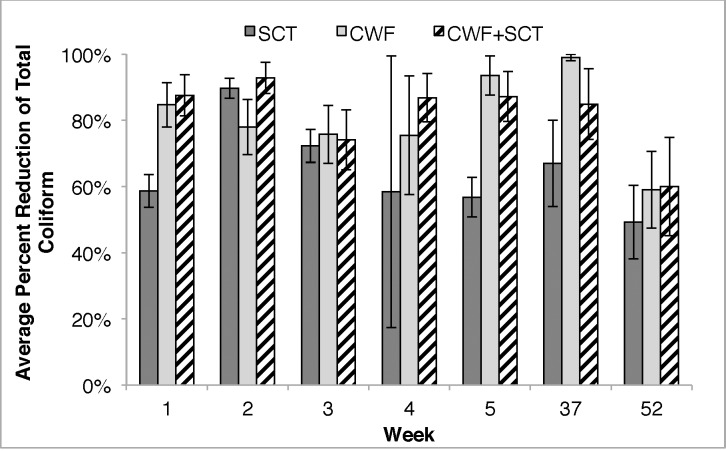
Average percent reduction of total coliform over time. Samples were treated with silver-embedded ceramic tablet (SCT), ceramic water purification systems (CWF) or ceramic water purification systems with the silver-embedded ceramic tablet (CWF+SCT). Percent reduction was calculated based off of control ceramic tablets (for SCT-treated samples) or source water (for CWF- and CWF+SCT-treated samples). Data points represent average and error bars represent standard error.

Fig 2 shows the effect of the three ceramic-based POUWTs on percent reduction of TC. For SCT households, percent reduction in bacteria was calculated by taking the difference in bacteria concentrations between the blind control and silver-embedded ceramic tablet-treated samples, and dividing by the control. As a primary POUWT method, SCT households achieved an average percent reduction in TC between 59–90% for weeks 1–5, 37 and 52. When used as a secondary POUWT with CWFs, reduction in TC was calculated by subtracting bacterial concentrations in the influent samples from those in the effluent and then dividing by the TC levels in the influent sample. No difference in average percent reduction of TC was seen between CWF and CWF+SCT.

Results in S2 Fig show median reductions during weeks 1–5 and 37 for CWF and CWF+SCT households. At week 52, the median reduction in TC declined to 99% for both CWF and CWF+SCT groups. S3 Fig shows the percent of households with at least 90% reduction in TC each week.

The efficiency of CWFs and CWF+SCT to reduce *E*. *coli* is displayed in S4, S5 and S6 Figs. Median percent removal of *E*. *coli* was 100% for weeks 1–5. At week 52, median percent reduction of *E*. *coli* was 99% for both groups (S4 Fig). A difference in percent reduction of *E*. *coli* was observed at week 52, with an average of 60% and 100% reduction in *E*. *coli* among CWF and CWF+SCT groups, respectively (S5 Fig). The distribution of high-performing ceramic water filter systems are shown in S6 Fig for *E*. *coli* reduction. At week 52, the CWF+SCT group had a higher number of CWF+SCTs providing 100% reduction in *E*. *coli* compared to the control group. The percent of households in the CWF group with 100% reduction in *E*. *coli* was very high ranging from 60% to 100% at weeks 1–5, 37 and 52. Among the CWF+SCT households, 100% of samples were completely free of *E*. *coli* at weeks 3,4, 37 and 52, (S6 Fig). S7 Fig shows average percent reduction in *E*.*coli* among SCT households. S3 Table also shows the geometric mean of bacterial reduction (total coliform and *E*.*coli*) for all three ceramic-based POUWTs over one year. Geometric mean was calculated among working filters for all three interventions. The same trend was seen among the POUWTs when comparing geometric mean of *E*.*coli* and total coliform reductions, bacterial reduction was consistently high among CWF and CWF+SCT groups. Furthermore, minimal difference was seen between the arithmetic (average) and geometric mean in total coliform and *E*.*coli* reduction as can be seen in S5 Fig, S3 Table and Fig 2.

S8 Fig shows median percent reduction in TC and *E*. *coli* in drinking water treated with SCTs over 12 months. The *E*. *coli* median reduction was 100% for weeks 1–3, 5 and 37, and dropped to 0% and 79% during weeks 4 and 57. The distribution of high-performing SCTs is shown in S9 and S10 Figs. The efficacy of SCT declines over time for both TC (S9 Fig) and *E*. *coli* (S10 Fig). None of the households had 100% reduction in TC at week 52. S10 Fig shows that the SCT completely disinfected *E*. *coli* (100%) among 83% and 93% of the houses in weeks 1 and 2. For weeks 37 and 52, 40% of samples were completely free of *E*. *coli*, and there was at least 80% reduction was observed among 60% at week 52. Table 1 summarizes the percent of SCT-, CWF- and CWF+SCT-treated samples that met WHO risk category requirements.

**Table 1:** WHO risk category of samples from households using SCT, CWF and CWF+SCT POU water treatment methods.

WHO Risk Category	*E*. *coli*	Week						
	(CFU/100mL)	1	2	3	4	5	37	52
**SCT**								
No risk	< 1	36%	74%	36%	29%	57%	40%	40%
Low risk	1 to 10	64%	26%	59%	53%	14%	0%	40%
Medium risk	11 to 100	0%	0%	5%	18%	29%	60%	20%
High risk	>100	0%	0%	0%	0%	0%	10%	0%
**CWF**								
No risk	<1	100%	87%	100%	100%	95%	100%	63%
Low risk	1–10	0%	9%	0%	0%	5%	0%	38%
Medium risk	11–100	0%	4%	0%	0%	0%	0%	0%
High risk	>100	0%	0%	0%	0%	0%	0%	0%
**CWF +SCT**								
No risk	<1	96%	73%	100%	100%	95%	100%	100%
Low risk	1–10	4%	5%	0%	0%	5%	0%	0%
Medium risk	11–100	0%	23%	0%	0%	0%	0%	0%
High risk	>100	0%	0%	0%	0%	0%	0%	0%

**Fig 3:**
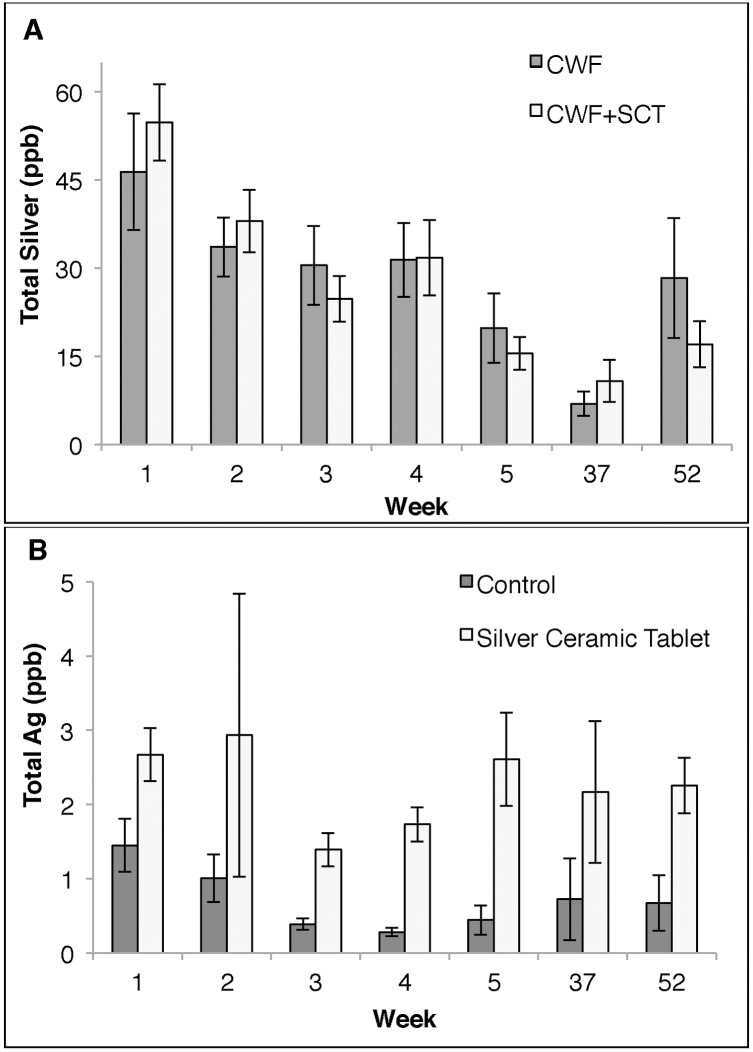
Total silver levels among water samples taken from households using ceramic water purification systems over 12 months. (A) Silver concentrations among households were either using the ceramic water purification system (CWF) or with the silver-embedded ceramic tablet (CWF+SCT). (B) Total silver concentrations among households with water storage containers with control and silver-embedded ceramic tablets. Data points represent average silver concentrations. Standard error was used to calculate error bars.

Total silver released into solution by the three PoUWT methods is shown in Fig 3. Silver released into solution was determined by subtracting silver concentrations in the source water sample from those in the effluent sample for CWF and CWF+SCT-treated samples. No significant difference was seen in average silver levels between the control and intervention group. For SCT households, total silver concentrations in water storage containers are shown in Fig 3. Average silver concentrations were higher among samples treated with the SCT compared to the control for all weeks except week 2 and 37 where no difference was seen.

**Fig 4:**
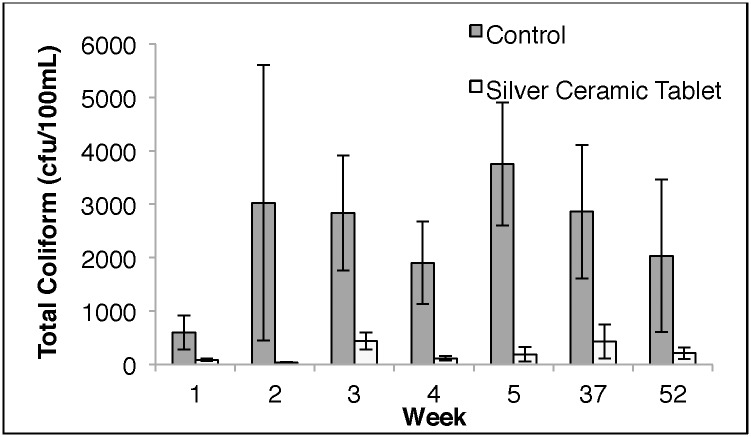
Total coliform bacteria in water storage containers with ceramic tablets. Control samples represent samples taken from the water storage container with the control ceramic tablet. Silver ceramic tablet samples represent samples taken from water storage containers with the silver-embedded ceramic tablet. Data points represent average total coliform levels among all households per week. Standard error was used to calculate error bars.

Bacterial load was evaluated by comparing TC levels in the control water storage containers to those in water storage containers with the silver-embedded ceramic tablet. Fig 4 compares disinfection efficiency relative to bacterial load in silver-embedded ceramic tablet-treated samples. TC concentrations remained consistently low in water storage containers when treated with silver-embedded ceramic tablets compared to the control. TC concentrations in the storage containers with the control tablet were always higher over one year.

S4 Table summarizes baseline TC concentrations and overall water quality for households using SCTs as their primary POU method. Baseline bacterial concentrations were determined by quantifying TC levels in samples treated with the control tablet each week. This represents the water quality over time at the household level when using a storage container. Water quality determined by comparing samples treated with control tablet and silver-embedded ceramic tablet. The water quality was considered ‘improved’ if TC levels were lower among samples treated with the silver-embedded ceramic tablet compared to the control. The water quality declined if TC concentrations were higher in silver-embedded ceramic tablet-treated samples than in the control. Water quality improved among majority of samples as shown in S4 Table.

S11 Fig shows the recontamination frequency of water at the household level over 5 weeks. Samples were collected at the water source of each household in the village and compared against the water quality in the control water storage container over 5 weeks.

### Comparing silver-embedded ceramic tablet field performance to laboratory

Ceramic tablets sampled at week 37 were removed from homes and brought back to the laboratory for further laboratory testing of silver release. Silver release levels from laboratory testing and field-testing are compared in S12 Fig. Silver concentrations were normalized against the blind control by subtracting silver concentrations in the blind control from those in the silver-embedded ceramic tablet-treated sample. Silver concentrations among samples treated with the silver-embedded ceramic tablet were higher in laboratory samples compared to field samples among all households with the exception of tablets from two houses. The average residual silver concentration of water treated with the silver-embedded ceramic tablet was 8.97±3.85 μg/L in the laboratory and 1.45±0.611 μg/L in the field.

### Effects of turbidity on ceramic water filters and silver-embedded ceramic tablet performance

Turbidity was measured among all the households during week 37 and 52. S13 Fig shows average turbidity levels among households using CWFs and CWF+SCTs. Turbidity was measured pre- and post-treatment. Average turbidity levels were higher among samples collected post-treatment (5.46±2.03, 3.58±0.86 NTU) compared to samples taken prior to any treatment (1.66±0.32, 2.28±0.59 NTU).

**Fig 5:**
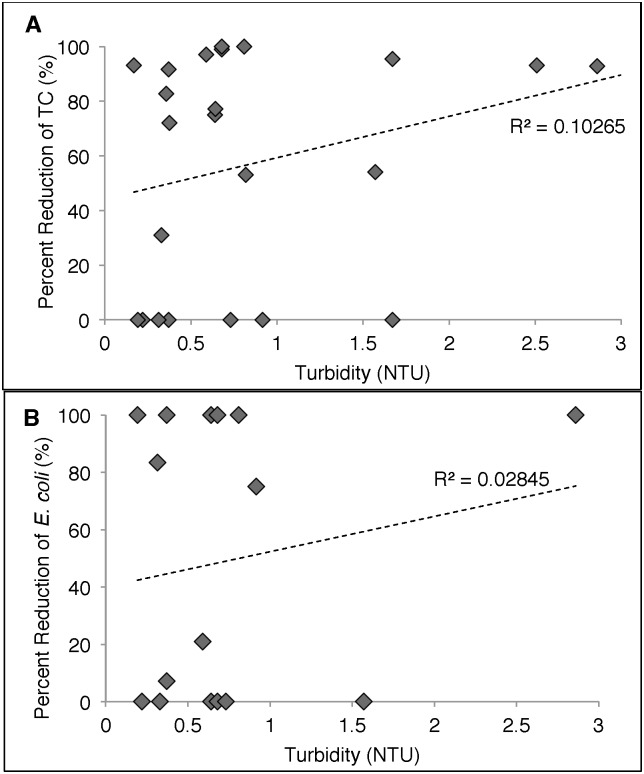
Correlation of turbidity and percent reduction in bacteria among households using the SCT method. Samples were treated with silver-embedded ceramic tablets and analyzed for reduction in total coliform bacteria (A) and E. coli (B) turbidity. Samples were taken at 37 and 52 weeks post-intervention.

There was no difference in turbidity levels among samples taken from CWF households and CWF+SCT households both pre- and post-treatment. For SCT households average turbidity was 0.888±0.176 NTU. In Fig 5 turbidity measurements were correlated to percent reduction in bacteria among households only using the silver-embedded ceramic tablet. Turbidity levels in samples treated with silver-embedded ceramic tablet, as the primary POU method, were plotted against corresponding percent reductions in TC (Fig 5A) and *E*. *coli* (Fig 5B). No correlation was observed between turbidity and disinfection efficiency. Linear regression was performed and r-squared values were 0.102 and 0.028 for percent reduction of TC and *E*. *coli* compared to turbidity, respectively, demonstrating the performance of silver-embedded ceramic tablet was unaffected by turbidity levels.

### Social acceptability

All participants preferred using the POU methods to their current POU methods. The only improvements participants suggested for ceramic water filters and silver-embedded ceramic tablets was to have them treat larger volumes of water at a given time. All participants said that the ceramic-based POU methods improved the taste and odor of their water. Prior to these interventions, 56% of participants stored water in plastic buckets or drums. Storage containers were stored indoors among 95% of households and 57% cleaned containers with soap. 32% of participants claimed to never clean the storage containers. Water practices and demographic data of participants are described in S5 and S6 Tables.

Willingness-to-pay (WTP) surveys were conducted among 79 households during the exit survey. Trends are shown in S14 and S15 Figs. Binning methods were used to determine WTP [19]. WTP prices for the SCT, CWF and CWF+SCT began at R40 ($3.40 USD), R130 ($11 USD) and R200 ($17 USD) and increased by R10 ($1 USD) or decreased by R5 ($0.5 USD) depending on the response of the participant. S14 Fig shows the percent of participants willing to pay for the silver-embedded ceramic tablet, ceramic water filters or CWF+SCTsystem at various price points. Approximately 50% of participants using the CWF and CWF+SCT were willing to pay between R100-R125 ($8–10 USD) and only R30 ($2.50 USD) for the SCT by itself. Median WTP for ceramic water filter units was R120 ($12 USD) and for ceramic water filters with the silver-embedded ceramic tablet R100 ($8 USD). Among households only using the silver-embedded ceramic tablet, 50% of households were willing to pay between R50 ($4.25 USD) and R55 ($4.70 USD) for silver-embedded ceramic tablets (S15 Fig). Median WTP for silver-embedded ceramic tablets was R50 ($4.25 USD) among households using the silver-embedded ceramic tablet as their primary POU method, and only R27.5 among households with the CWF+SCTsystem.

## Discussion

This paper evaluates the performance of three ceramic-based PoUWT technologies: silver-embedded ceramic tablets, ceramic water filters and a filter-tablet combination (Fig 1 and S1 Fig). Short-term performance of all three PoUWT methods were consistent with findings of previous studies [7,20,21]. Households using ceramic water filters and those using the filter-tablet combination had consistently high reductions in bacteria, demonstrating 86% and 99% average percent reduction in *E*. *coli* after 5 weeks (S5 Fig). Early performances of the silver-embedded ceramic tablet as the stand-alone PoUWT method demonstrated average reductions in *E*. *coli* (93%) that are comparable to existing PoUWT methods, such as ceramic water filters (79%, 97.5%) [14], the filter-tablet combination(90%), chlorination (93.7%) [7] and flocculent/disinfectant powders (89.53%) [7]. This suggests that the silver-embedded ceramic tablet as the stand-alone PoUWT method may be just as effective as other PoUWT methods.

However, the silver-embedded ceramic tablet performance did decline over time, and this may be due to limitations in the study, which included lesser than expected silver release amounts, low baseline *E*. *coli* levels and small sample size. Even with these limitations though, the performance of the silver-embedded ceramic tablet is comparable to other PoUWT methods. Albert et al [7] evaluated ceramic water filters, Waterguard (Chlorine-based liquid) and PUR (flocculent/disinfection powder) for two months and found 39%, 51%, and 33% of treated samples had <1 CFU /100mL of *E*. *coli*, respectively. Among households using the silver-embedded ceramic tablet as the stand alone PoUWT method, a higher percentage of treated-samples (57%) had < 1 CFU/100mL of *E*. *coli* after 5 weeks.

Many PoUWT intervention studies have been limited to short-term evaluations of field performance due to cost, time and resources. Only a few studies have evaluated long-term efficacy and these studies have generally shown a decline in performance [9,20,21]. Ceramic water filters and biosand filters have been considered some of the most effective longterm PoUWTs. However, these technologies require adequate maintenance and constant training on proper usage and repair [9,10,22]. For example, Sisson et al (2013) found biosand filters to be working after 12 years in Haiti, however only 47% of the original 55 biosand filters were still functioning. They found that incentives to use the filters and ability to repair filters contributed to the decline in use. Thus long-term field-testing of PoUWT methods are essential to ensure their performance and impact on health. In a paper published by Mellor et al [23], safe drinking water was correlated to community health that demonstrated improvements in human health are achieved when PoUWT interventions provide at least a 3-log reduction in total coliform consistently over 3 years.

In this study, long-term performance of all three ceramic-based PoUWT interventions were evaluated to better understand the human health impact of the silver-embedded ceramic tablet as a PoUWT. Ceramic water filter performance was comparable to previous long-term ceramic water filter intervention studies [4,5,14,23]. Households using the silver-embedded ceramic tablet as the stand-alone PoUWT method observed a decline in microbial disinfection compared to households using ceramic water filters. However silver-embedded ceramic tablet long-term performance was consistent with other low-cost, single-use PoUWT methods, such as chlorination. Overall, the common trend was as expected, the PoUWT method with the highest microbial disinfection efficiency was the filter-tablet combination, followed by the ceramic water filters, and then the stand-alone silver-embedded ceramic tablet intervention. Median percent reduction of TC and *E*. *coli* were high among all three interventions, ranging between 90–100% over one year.

In addition to challenges in testing PoUWT longevity, there can be challenges of high variability between samples and bias in user compliance when conducting field studies. Only chlorine-based PoUWT methods have been eligible for blinded field studies [11,24–26], however these studies may not represent a truly blinded test because of the residual taste of chlorine in chlorine-treated water. Until now, these challenges could not be accounted for due to a lack of a true blind control for many PoUWT methods. Given the nature of the silver-embedded ceramic tablet, a control ceramic tablet can be developed that has no silver however looks identical to the silver-embedded ceramic tablet. The silver embedded within the ceramic tablet is not visible to the human eye. Thus, a true blinded study was conducted to evaluate the silver-embedded ceramic tablet as the stand-alone PoUWT method. The performance of the silver-embedded ceramic tablet was compared against the blinded control in each household. Both tablets were used in parallel in each household reducing user bias and variability in water quality among control and intervention samples. In comparison to a blinded, randomized control trial evaluating sodium dichloroisocyanurate (NaDCC) tablets, the silver-embedded ceramic tablet performed better, with 40% of treated samples with < 1CFU/100mL of *E*. *coli* compared to 37% seen in the study by Boisson et al [11].

As for the ceramic water filters in this study, their performance was comparable to other long-term ceramic water filter studies. Clasen and Boisson [27] observed that 54% of households with working ceramic candle filters were free of thermototal coliform after 16 mos. This is consistent with the findings in this paper, where 63% ceramic water filter-treated samples had < 1 CFU/100mL of *E*. *coli* after 1 year. Average percent reduction of *E*. *coli* among ceramic water filter households declined to 60% after 52 weeks, which is lower than what has previously been seen in long-term ceramic water filter studies. Kallman et al [14] and Mellor et al [5], observed 92% and 95.7% reduction in *E*. *coli* among households using locally made ceramic water filters in Guatemala after a year.

Potential inconsistencies in these results may be due to different baseline *E*. *coli* levels in each study. Mean baseline levels were 163 CFU/100mL [14] and 269 CFU/100mL [5] in the Guatemala, while in S. Africa they were 17 CFU/100mL. Also, there may have been differences in the quality of filters. Filters were produced at two different factories that may have had slightly different manufacturing methods. Also, the different factories use different types of clay and raw materials, lending to potential differences in filter performance. It has been shown that the predominant clay mineral for Guatemalan ceramic water filters was illite, and smectite for the S. African ceramic water filters [6,17]. The differences in clay mineralogy may contribute to variations in the durability of ceramic water filters. Over time, the structure of the S. African ceramic water filter showed signs of deterioration. The ceramic media in S. African ceramic water filters became more fragile and clay residue was observed in the effluent. As a result, turbidity was higher in effluent samples (S13 Fig) and the shape of the ceramic water filter had slightly altered, loosening its fit in the lower reservoir and making the treated-water more prone to contamination. Ceramic water filters produced in Guatemala may have been sturdier, due to the properties of the clay, and thus maintaining high performances.

The filter-tablet combination PoUWT method performed the best out of all the PoUWT methods. All filter-tablet combination-treated samples were free of *E*. *coli* after 1 year, and average percent reduction in *E*. *coli* was 100%. This is higher than what was observed among households using ceramic water filters, suggesting that the secondary treatment by the silver-embedded ceramic tablet improved the microbiological performance of ceramic water filters. The only other study to our knowledge that has evaluated a secondary PoUWT method with ceramic water filters is a study by Mellor et al [5] where a silver-impregnated ceramic torus was placed in the lower reservoirs of ceramic water filters in households in Guatemala. The ceramic torus was developed by PFP and produced exactly the same way as PFP ceramic water filters. It was designed to release silver into solution to provide continual disinfection. After one year, no significant difference was observed in microbial disinfection or silver release between ceramic water filters with (96.9%) or without the PFP ceramic torus (95.7%). In results presented in this paper, a difference in *E*. *coli* reduction was observed at week 52 between control and intervention groups. The difference in performance could be because only a small fraction (~10%) of the silver embedded in the silver-embedded ceramic tablet was embedded in the torus ceramic, therefore the torus ceramic PoUWT was not an effective PoUWT method compared to the silver-embedded ceramic tablet. The silver-embedded ceramic tablet was evaluated as a primary PoUWT method in addition to a secondary PoUWT method in this study, and it was shown that the silver-embedded ceramic tablet does disinfect waterborne pathogens through the release of silver (Figs 2 and 4) among water storage containers and reduce recontamination of stored water (Fig 2). To our knowledge, studies to this extent have not been conducted on the PFP ceramic torus.

Neither study observed differences in average residual silver concentrations after 52 weeks. However, this may not have been reflective of microbial disinfection efficiency. For the PFP ceramic torus, it is not evident if silver release was in sufficient quantities or in the right form of silver to provide microbial disinfection [5]. For filter-tablet combination-households, silver release in the treated water was a combination of silver washing of the ceramic water filter and silver being released by the silver-embedded ceramic tablet. One potential explanation for this discrepancy in microbial disinfection and silver release data in this study may be that differences in silver concentrations are more representative of silver washing off the filters than silver being released by the silver-embedded ceramic tablet. Fig 3A demonstrates that average residual silver concentrations in ceramic water filter-treated samples ranged from 46.4 μg/L to 6.94 μg/L, while for water samples treated with the stand alone silver-embedded ceramic tablet the total silver concentrations ranged from 2.93 μg/L to 1.39 μg/L (Fig 3B). The variation in silver washing-off the ceramic water filter was greater than the variation in the silver being released from silver-embedded ceramic tablets. Thus the variation in total residual silver concentrations may be more representative of different amounts of silver leaching off the filters than representative of additional silver being released into solution due to the presence of silver-embedded ceramic tablets.

Also it is important to note that silver released from the ceramic water filters and PFP ceramic torus is most likely in the metallic form, since colloidal silver was used in their production. Majority of the silver released from silver-embedded ceramic tablet has been shown to be in the ionic form [17]. Furthermore, previous studies have demonstrated that ionic silver is a stronger disinfecting agent than colloidal silver [28]. Therefore the filter-tablet combination and ceramic water filter-treated samples may have had different ratios of ionic to metallic silver in solution, which may impact microbial disinfection efficiency. If so, then differences would not been observed in total silver concentration, but reflected in microbial disinfection. This provides another explanation as to why the PFP ceramic torus may not have been effective. If any silver is being released from the ceramic torus, it is most likely in the metallic form and therefore less effective as a microbial disinfectant compared to the ionic silver released from the silver-embedded ceramic tablet. Of course, silver levels among silver-embedded ceramic tablet-treated samples are very low therefore further testing needs to be done to validate this hypothesis, but observations from this study suggest the silver-embedded ceramic tablet provided continual disinfection through the release of ionic silver, which was effective in improving ceramic water filter performance at week 52.

During this field study, there were a few limitations. One major limitation was the silver release rates of ceramic tablets at lower than expected levels. In laboratory experiments, the silver-embedded ceramic tablet was shown to release 26 μg/L after 24 hours in 10 L of phosphate buffer solution [17]. The same silver concentrations were expected among silver-embedded ceramic tablet-treated field samples, however silver concentrations were much lower, ranging from 2.93 μg/L to 1.39 μg/L. To determine if the low silver levels were due to the performance of the silver-embedded ceramic tablet or due to user compliance, silver-embedded ceramic tablet were collected from homes visited at week 37 and tested for silver release in a controlled laboratory setting. Silver levels were higher in samples collected in the laboratory compared to the field, however overall mean silver levels were lower than what was previously observed in the lab. One potential explanation of the different silver levels between field and lab samples may be due to treatment times of silver-embedded ceramic tablets in households prior to collection. In the laboratory, water was treated for exactly 24 h with the silver-embedded ceramic tablet. In the field, participants were instructed to always keep the ceramic tablet in their storage containers and allow at least 8 h for treatment of fresh water. These treatment times were not monitored directly in the field therefore lower silver concentrations in the field may be reflective of different exposure times of the silver-embedded ceramic tablet. Furthermore, different water chemistries and pH levels may impact silver release by affecting oxidation rates of metallic silver [29]. Also, the presence of dissolved organic matter in natural water may impact surface chemistries of silver nanopatches and thus hindering silver release. Future work includes investigating the fundamental chemical processes that are regulating the organic formation of the silver nanopatches.

Overall, this study provides support for both the short-term and long-term effectiveness of three ceramic-based PoUWT methods. It provides a non-biased evaluation of a novel PoUWT method, silver-embedded ceramic tablets. Results suggest this novel PoUWT method is effective in improving water quality. Performance of the silver-embedded ceramic tablets among households using it as their primary PoUWT method demonstrates that the silver-embedded ceramic tablets is just as effective in improving water quality compared to other low-cost, portable methods such as chlorine and flocculent/disinfecting powders. Survey data suggests that the silver-embedded ceramic tablets are also socially acceptable in comparison to other low-cost, single-use methods due to their reusability, ease of use and ability retain the natural taste and odor of water. Even at low residual silver concentrations, the performance of the silver-embedded ceramic tablet as a stand-alone PoUWT method is comparable to ceramic water filters systems, as seen at week 2 in Figs 2 and 3. Long-term performance is best achieved with the filter-tablet combination, where the silver-embedded ceramic tablet helps reduce recontamination of treated water. Future work includes modeling the oxidation and diffusion rates of silver nanopatche formation and silver release, investigating effects of various water qualities and chemistries on silver release. Also additional work is being done on material composition to enhance diffusion of silver ions through porous structure. Future research should work to improve the silver release kinetics of the SCT and to better understand the role of water chemistry on silver oxidation and release from the porous ceramic matrix.

## Supporting Information

S1 Fig Outline of study to evaluate the technological performance of silver-embedded ceramic tablets, ceramic water filters, and ceramic water filters with ceramic tablets in the lower water reservoir in Limpopo Province, South Africa. (PDF)

S2 Fig Median percent reduction of total coliform bacteria over 12 months. The CWF group consisted of 25 households with only ceramic water purification systems. The CWF+SCT group consisted of 25 households with the ceramic water purification system and silver embedded ceramic tablet.
(PDF)

S3 Fig Percent of households with at least 90% reduction in total coliform bacteria over 12 months. Samples were taken from CWF households and households using the ceramic water purification system with a silver-embedded ceramic tablet (CWF+SCT).
(PDF)

S4 Fig Median percent reduction of *E*. *coli* among households using ceramic water purification systems over 12 months. 25 households were using ceramic water purification systems (CWF). 25 households were using the ceramic water purification system with the silver-embedded ceramic tablet (CWF+SCT).
(PDF)

S5 Fig Average percent reduction of *E*. *coli* over time. Households were using ceramic water purification systems (CWF) or ceramic water purification systems with the silver-embedded ceramic tablet (CWF+SCT). Data points represent average and error bars represent standard error.
(PDF)

S6 Fig Percent of households with 100% reduction in *E*. *coli* over time. Households were using ceramic water purification systems (CWF) or ceramic water purification systems with the silver-embedded ceramic tablet (CWF+SCT).
(PDF)

S7 Fig Percent reduction of *E*. *coli* over time. Households were using silver-embedded ceramic tablet (SCT) as primary water purification method. Data points represent average and error bars represent standard error. Percent reduction was determined by comparing *E*.*coli* concentrations in water storage containers treated with control ceramic tablets to those treated with silver-embedded ceramic tablets. Samples were taken in duplicate among 29 households over 12 months. Data points represent the median of all samples.
(PDF)

S8 Fig Median percent reduction of total coliform bacteria and *E*. *coli* among ceramic tablet treated samples. Percent reduction was determined by comparing bacteria levels in water storage containers treated with control ceramic tablets to those treated with silver-embedded ceramic tablets. Samples were taken in duplicate among 29 households over 12 months. Data points represent the median of all samples.
(PDF)

S9 Fig Percent of ceramic tablet-treated samples with at least 80, 90 and 100% reduction in total coliform bacteria over 12 months. (PDF)

S10 Fig Percent of ceramic tablet-treated samples with at least 80, 90 and 100% reduction in *E*. *coli* over 12 months. (PDF)

S11 Fig Total coliform bacteria levels at the water source and in water storage containers at the household level over time. The water storage containers that were sampled had the control ceramic tablet that did not have silver (Control). Data points represent average total coliform bacteria of all samples taken each week. Error bars represent standard error.
(PDF)

S12 Fig Field and laboratory analysis of silver concentrations ceramic tablet-treated samples after 37 weeks. Control and silver embedded ceramic tablets were used to treat 10 L of water among households for 37 weeks. Samples were taken at 37 weeks from 10 households, and ceramic tablets were reanalyzed in laboratory settings. Silver concentrations were normalized by subtracting silver levels in the control from those in silver-embedded ceramic tablet-treated samples. Laboratory samples were collected after 24 h of treatment. Average silver concentrations were calculated for samples taken at 37 weeks both in the field and laboratory. Standard error was used to represent error bars.
(PDF)

S13 Fig Turbidity of ceramic-based technologies. Turbidity levels pre- and post-treatment among houses using ceramic water purification systems. Pre-treatment represented by influent samples and post-treatment samples represented by effluent samples. Data points represent average turbidity levels determined at weeks 37 and 52 combined. Standard error is used to calculate error bars.
(PDF)

S14 Fig Willingness-to-pay for each POU intervention (SCT, CWF and CWF+SCT) among 79 households in Limpopo Province, S. Africa. WTP was determined using binning method.
(PDF)

S15 Fig Comparison of willingness-to-pay for SCT among households using SCT as primary POU method (SCT) and as secondary POU method (CWF+SCT). (PDF)

S1 Table Number of Households sampled each week in CWF-only and CWF-SCT households. (PDF)

S2 Table Number of houses visited and samples collected each week among SCT-only households. (PDF)

S3 Table Geometric mean of bacterial reduction among all three water purification interventions each week. (PDF)

S4 Table Baseline total coliform (TC) bacteria levels and water quality of samples among SCT households. (PDF)

S5 Table Demographic Data. (PDF)

S6 Table Water practices. (PDF)
