# Supplementary figures and images for: Evaluation of a Silver-Embedded Ceramic Tablet as a Primary and Secondary Point-of-Use Water Purification Technology in Limpopo Province, S. Africa
Source: PLoS One. 2017 Jan 17;12(1):e0169502. doi: 10.1371/journal.pone.0169502 (PMC5240968; doi:10.1371/journal.pone.0169502)

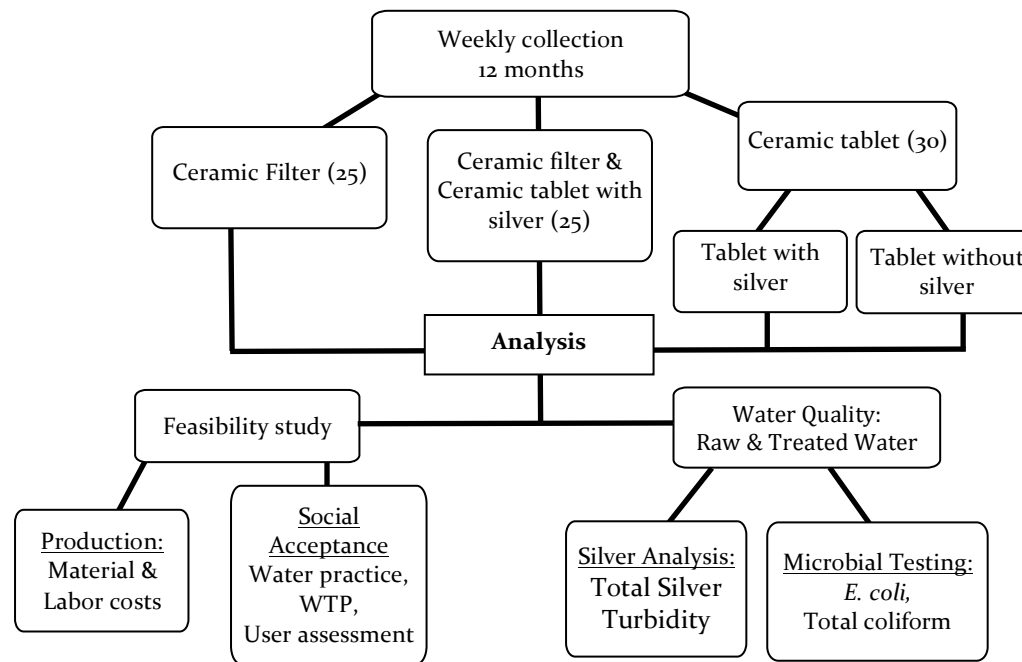

**S1 Fig. Outline of study to evaluate the technological performance of POU methods.**

Supplement: S1 Fig — (PDF) [file pone.0169502.s001.pdf]
