# Supplementary material for: Evaluation of a Silver-Embedded Ceramic Tablet as a Primary and Secondary Point-of-Use Water Purification Technology in Limpopo Province, S. Africa
Source: PLoS One. 2017 Jan 17;12(1):e0169502. doi: 10.1371/journal.pone.0169502 (PMC5240968; doi:10.1371/journal.pone.0169502)
Supplement: S2 Fig — The CWF group consisted of 25 households with only ceramic water purification systems. The CWF+SCT group consisted of 25 households with the ceramic water purification system and silver embedded ceramic tablet. (PDF) [file pone.0169502.s002.pdf]

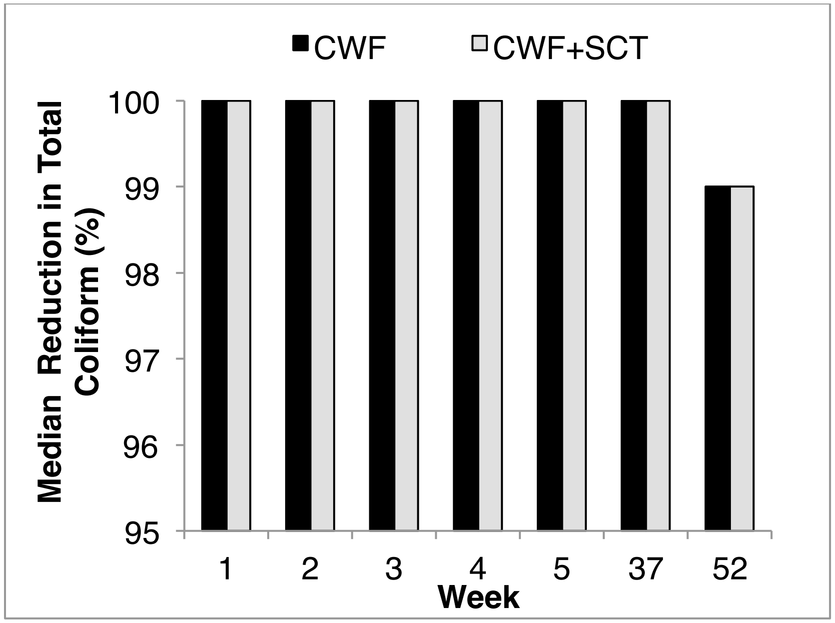

S2 Fig. Median percent reduction of total coliform bacteria over 12 months.
The CWF group consisted of 25 households with only ceramic water purification systems. The CWF+SCT group consisted of 25 households with the ceramic water purification system and silver embedded ceramic tablet.
