# Supplementary material for: Evaluation of a Silver-Embedded Ceramic Tablet as a Primary and Secondary Point-of-Use Water Purification Technology in Limpopo Province, S. Africa
Source: PLoS One. 2017 Jan 17;12(1):e0169502. doi: 10.1371/journal.pone.0169502 (PMC5240968; doi:10.1371/journal.pone.0169502)
Supplement: S3 Fig — Samples were taken from CWF households and households using the ceramic water purification system with a silver-embedded ceramic tablet (CWF+SCT). (PDF) [file pone.0169502.s003.pdf]

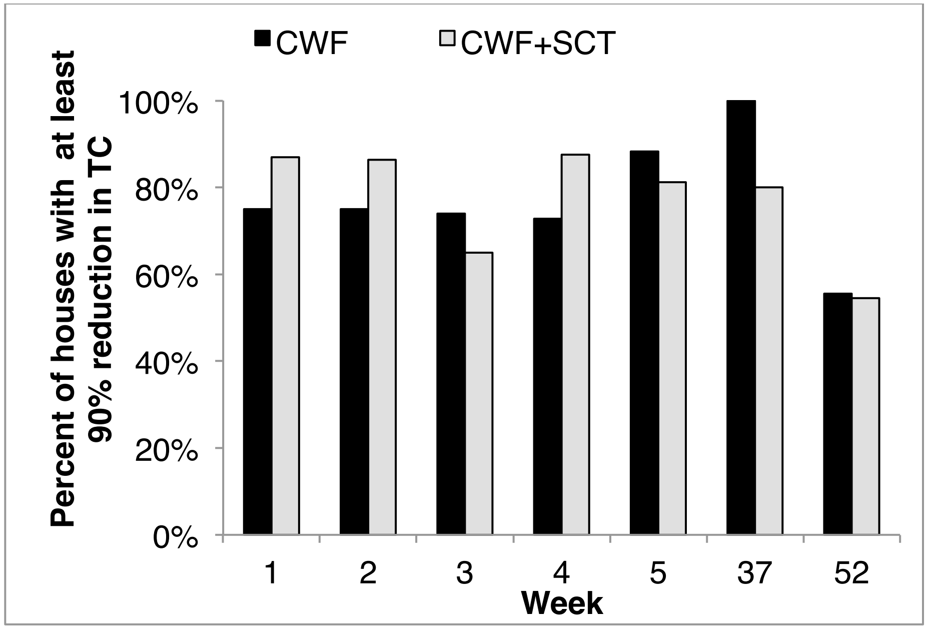

S3 Fig. Percent of households with at least 90% reduction in total coliform bacteria over 12 months.
Samples were taken from CWF households and households using the ceramic water purification system with a silver-embedded ceramic tablet (CWF+SCT).
