# Supplementary material for: Evaluation of a Silver-Embedded Ceramic Tablet as a Primary and Secondary Point-of-Use Water Purification Technology in Limpopo Province, S. Africa
Source: PLoS One. 2017 Jan 17;12(1):e0169502. doi: 10.1371/journal.pone.0169502 (PMC5240968; doi:10.1371/journal.pone.0169502)
Supplement: S4 Fig — 25 households were using ceramic water purification systems (CWF). 25 households were using the ceramic water purification system with the silver-embedded ceramic tablet (CWF+SCT). (PDF) [file pone.0169502.s004.pdf]

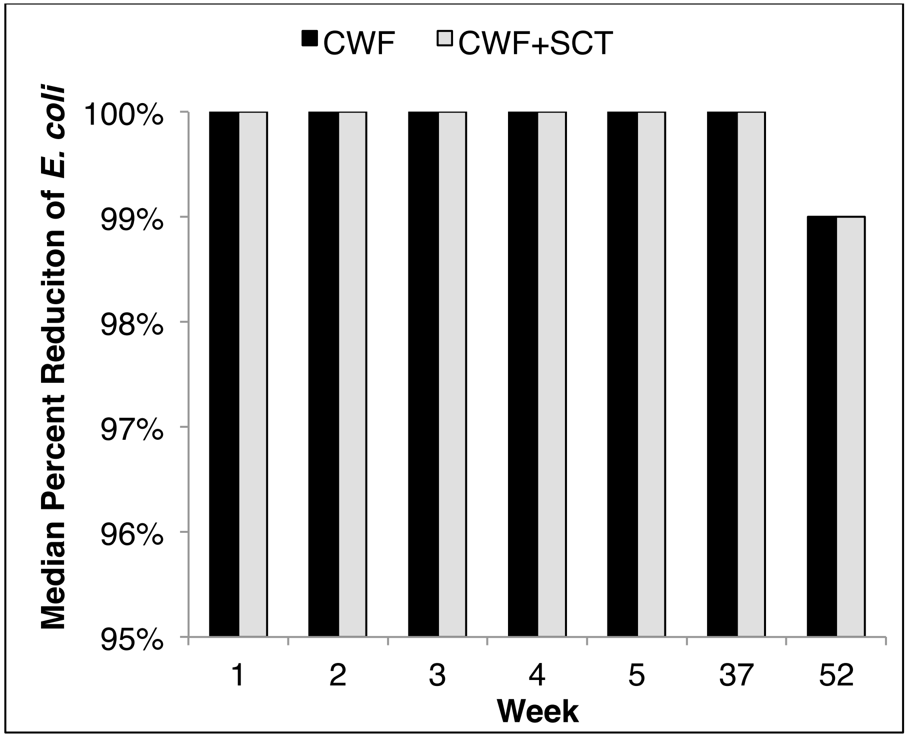

S4 Fig. Median percent reduction of *E. coli* among households using ceramic water purification systems over 12 months.
25 households were using ceramic water purification systems (CWF). 25 households were using the ceramic water purification system with the silver-embedded ceramic tablet (CWF+SCT).
