# Supplementary material for: Evaluation of a Silver-Embedded Ceramic Tablet as a Primary and Secondary Point-of-Use Water Purification Technology in Limpopo Province, S. Africa
Source: PLoS One. 2017 Jan 17;12(1):e0169502. doi: 10.1371/journal.pone.0169502 (PMC5240968; doi:10.1371/journal.pone.0169502)
Supplement: S5 Fig — Households were using ceramic water purification systems (CWF) or ceramic water purification systems with the silver-embedded ceramic tablet (CWF+SCT). Data points represent average and error bars represent standard error. (PDF) [file pone.0169502.s005.pdf]

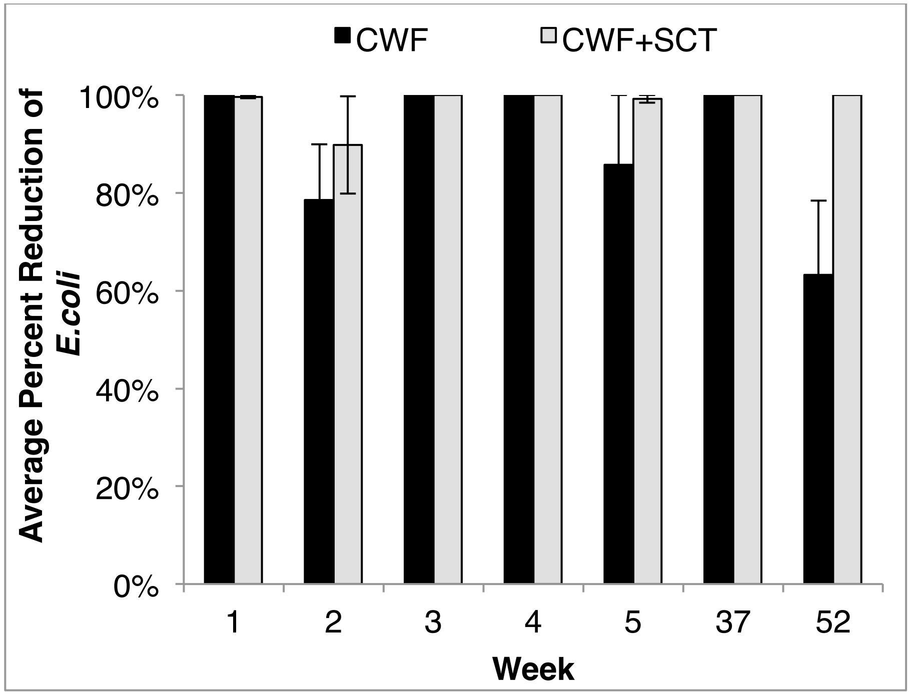

S5 Fig. Average percent reduction of *E. coli* over time. Households were using ceramic water purification systems (CWF) or ceramic water purification systems with the silver-embedded ceramic tablet (CWF+SCT). Data points represent average and error bars represent standard error.
