# Supplementary material for: Evaluation of a Silver-Embedded Ceramic Tablet as a Primary and Secondary Point-of-Use Water Purification Technology in Limpopo Province, S. Africa
Source: PLoS One. 2017 Jan 17;12(1):e0169502. doi: 10.1371/journal.pone.0169502 (PMC5240968; doi:10.1371/journal.pone.0169502)
Supplement: S6 Fig — Households were using ceramic water purification systems (CWF) or ceramic water purification systems with the silver-embedded ceramic tablet (CWF+SCT). (PDF) [file pone.0169502.s006.pdf]

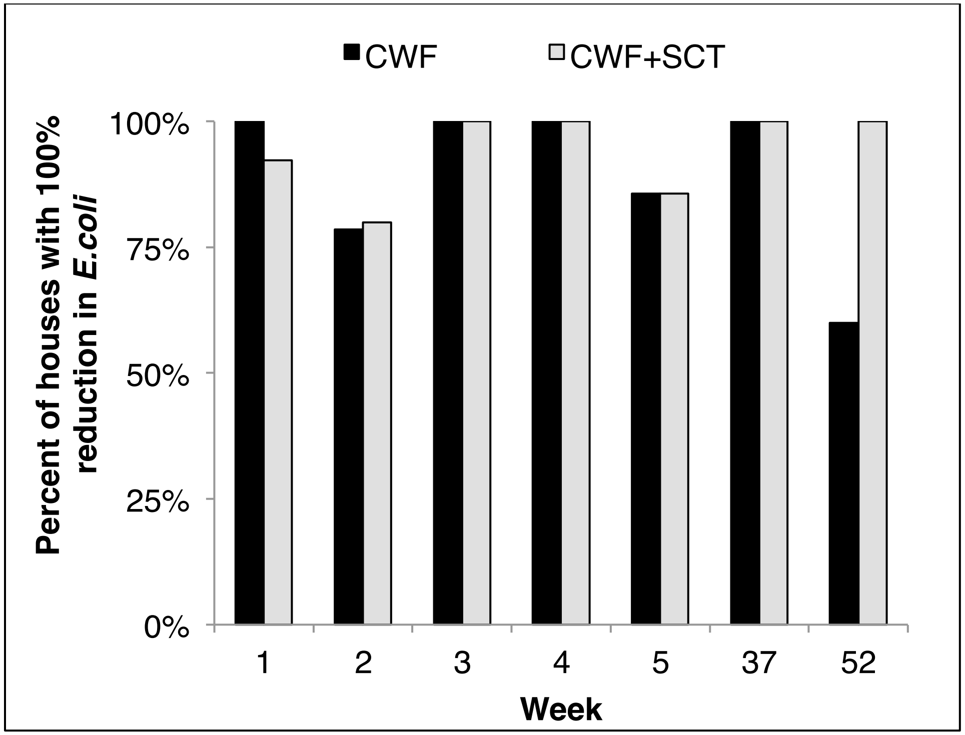

S6 Fig. Percent of households with 100% reduction in *E. coli* over time.
Households were using ceramic water purification systems (CWF) or ceramic water purification systems with the silver-embedded ceramic tablet (CWF+SCT).
