# Supplementary material for: Evaluation of a Silver-Embedded Ceramic Tablet as a Primary and Secondary Point-of-Use Water Purification Technology in Limpopo Province, S. Africa
Source: PLoS One. 2017 Jan 17;12(1):e0169502. doi: 10.1371/journal.pone.0169502 (PMC5240968; doi:10.1371/journal.pone.0169502)
Supplement: S7 Fig — Data points represent average and error bars represent standard error. Percent reduction was determined by comparing E.coli concentrations in water storage containers treated with control ceramic tablets to those treated with silver-embedded ceramic tablets. Samples were taken in duplicate among 29 households over 12 months. Data points represent the median of all samples. (PDF) [file pone.0169502.s007.pdf]

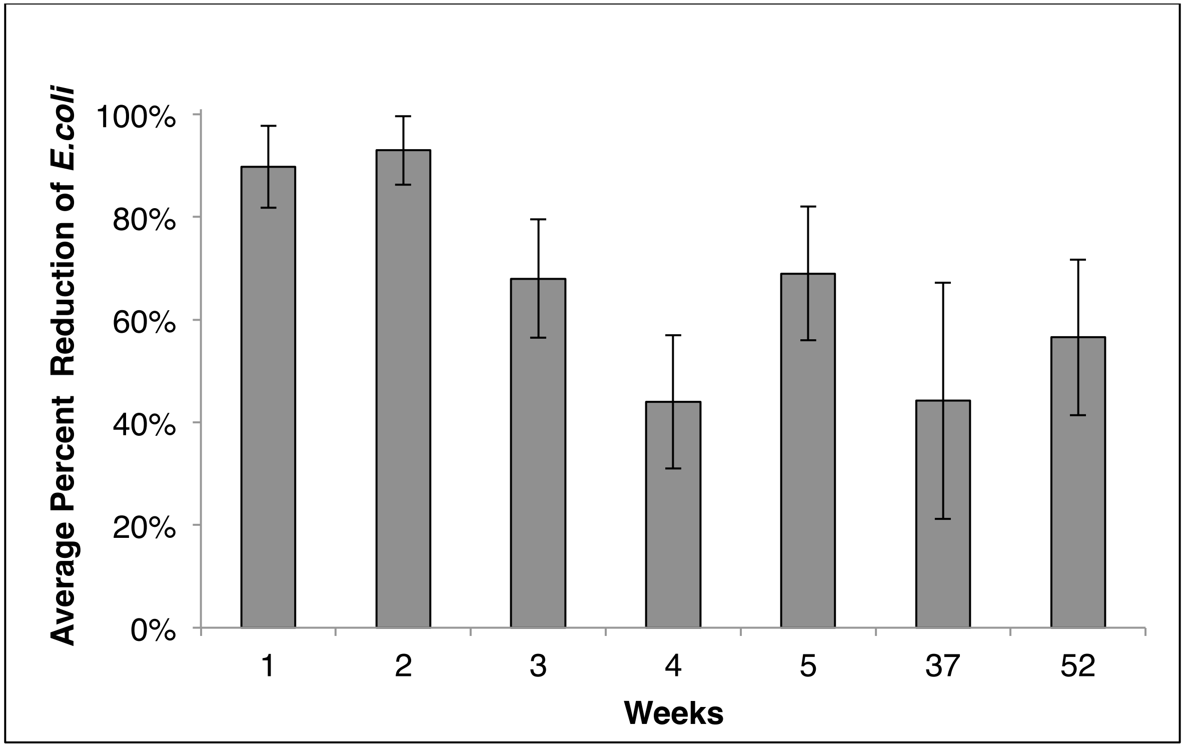

S7 Fig. Percent reduction of *E. coli* over time. Households were using silver-embedded ceramic tablet (SCT) as primary water purification method.

Data points represent average and error bars represent standard error. Percent reduction was determined by comparing *E.coli* concentrations in water storage containers treated with control ceramic tablets to those treated with silver-embedded ceramic tablets. Samples were taken in duplicate among 29 households over 12 months. Data points represent the median of all samples.
