# Supplementary material for: Evaluation of a Silver-Embedded Ceramic Tablet as a Primary and Secondary Point-of-Use Water Purification Technology in Limpopo Province, S. Africa
Source: PLoS One. 2017 Jan 17;12(1):e0169502. doi: 10.1371/journal.pone.0169502 (PMC5240968; doi:10.1371/journal.pone.0169502)
Supplement: S8 Fig — Percent reduction was determined by comparing bacteria levels in water storage containers treated with control ceramic tablets to those treated with silver-embedded ceramic tablets. Samples were taken in duplicate among 29 households over 12 months. Data points represent the median of all samples. (PDF) [file pone.0169502.s008.pdf]

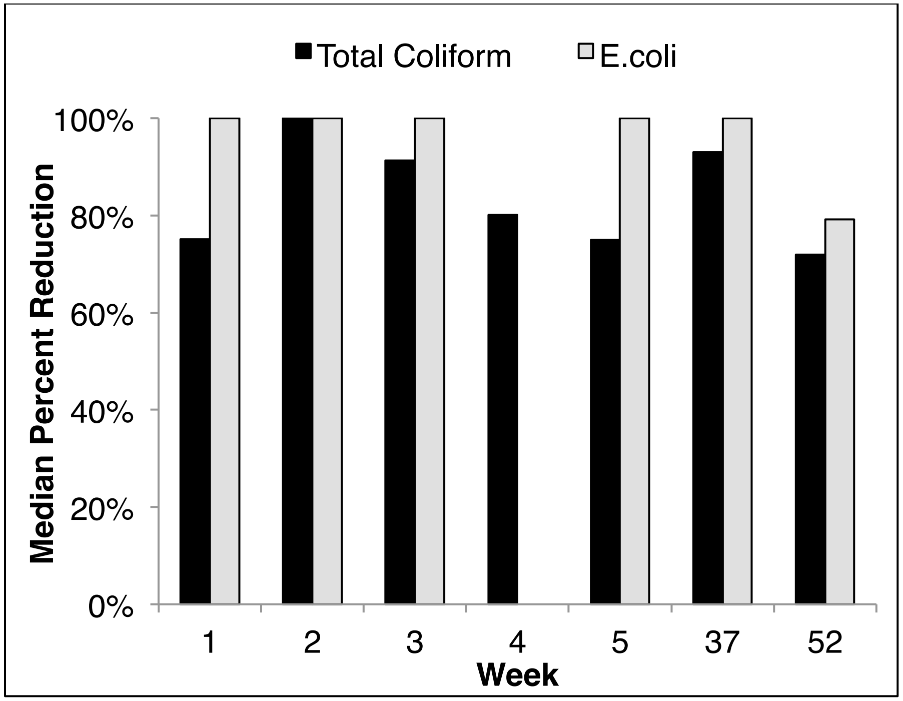

S8 Fig. Median percent reduction of total coliform bacteria and *E. coli* among ceramic tablet treated samples. Percent reduction was determined by comparing bacteria levels in water storage containers treated with control ceramic tablets to those treated with silver-embedded ceramic tablets. Samples were taken in duplicate among 29 households over 12 months. Data points represent the median of all samples.
