# Supplementary material for: Evaluation of a Silver-Embedded Ceramic Tablet as a Primary and Secondary Point-of-Use Water Purification Technology in Limpopo Province, S. Africa
Source: PLoS One. 2017 Jan 17;12(1):e0169502. doi: 10.1371/journal.pone.0169502 (PMC5240968; doi:10.1371/journal.pone.0169502)
Supplement: S11 Fig — The water storage containers that were sampled had the control ceramic tablet that did not have silver (Control). Data points represent average total coliform bacteria of all samples taken each week. Error bars represent standard error. (PDF) [file pone.0169502.s011.pdf]

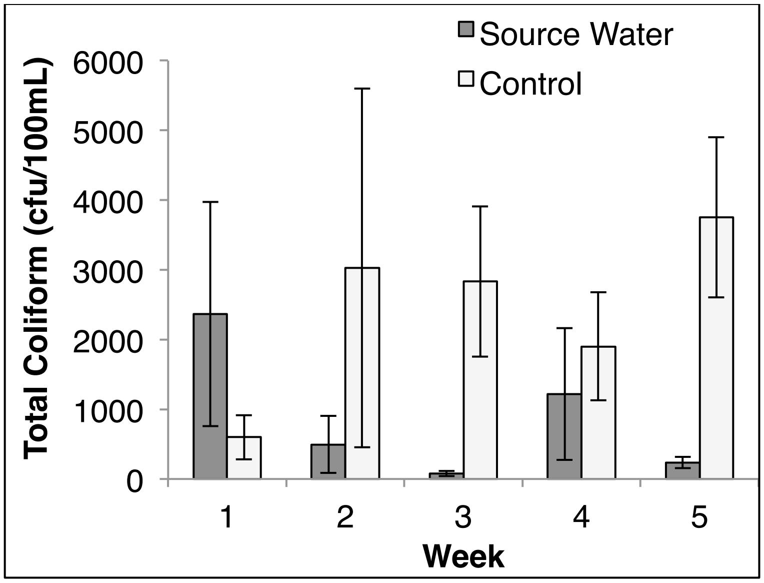

S11 Fig. Total coliform bacteria levels at the water source and in water storage containers at the household level over time.

The water storage containers that were sampled had the control ceramic tablet that did not have silver (Control). Data points represent average total coliform bacteria of all samples taken each week. Error bars represent standard error.
