# Supplementary material for: Evaluation of a Silver-Embedded Ceramic Tablet as a Primary and Secondary Point-of-Use Water Purification Technology in Limpopo Province, S. Africa
Source: PLoS One. 2017 Jan 17;12(1):e0169502. doi: 10.1371/journal.pone.0169502 (PMC5240968; doi:10.1371/journal.pone.0169502)
Supplement: S12 Fig — Control and silver embedded ceramic tablets were used to treat 10 L of water among households for 37 weeks. Samples were taken at 37 weeks from 10 households, and ceramic tablets were reanalyzed in laboratory settings. Silver concentrations were normalized by subtracting silver levels in the control from those in silver-embedded ceramic tablet-treated samples. Laboratory samples were collected after 24 h of treatment. Average silver concentrations were calculated for samples taken at 37 weeks both in the field and laboratory. Standard error was used to represent error bars. (PDF) [file pone.0169502.s012.pdf]

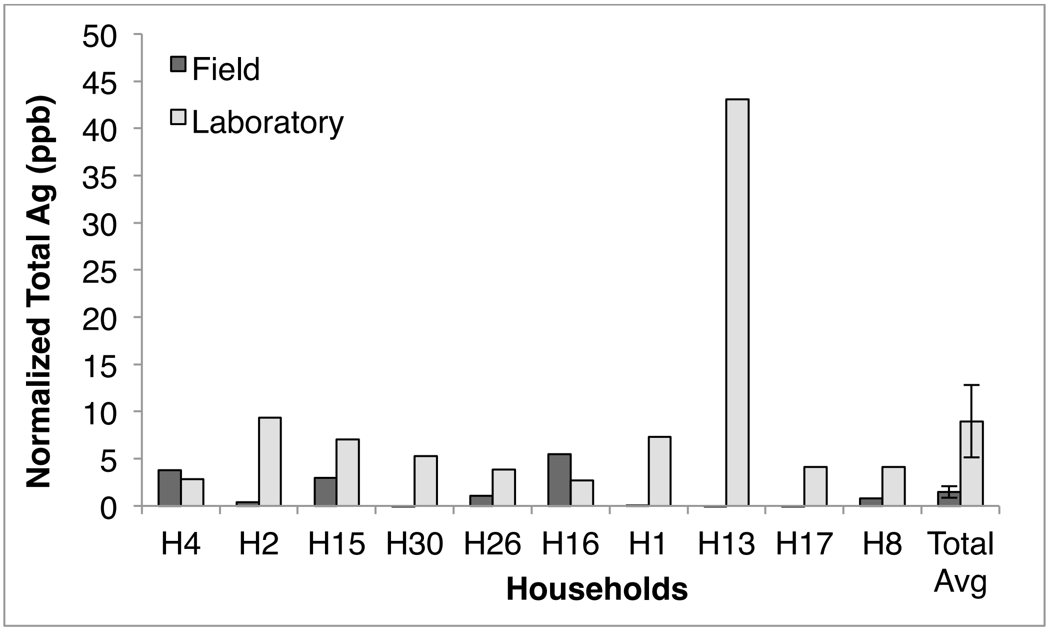

S12 Fig. Field and laboratory analysis of silver concentrations ceramic tablet-treated samples after 37 weeks.

Control and silver embedded ceramic tablets were used to treat 10 L of water among households for 37 weeks. Samples were taken at 37 weeks from 10 households, and ceramic tablets were reanalyzed in laboratory settings. Silver concentrations were normalized by subtracting silver levels in the control from those in silver-embedded ceramic tablet-treated samples. Laboratory samples were collected after 24 h of treatment. Average silver concentrations were calculated for samples taken at 37 weeks both in the field and laboratory. Standard error was used to represent error bars.
