# Supplementary material for: Evaluation of a Silver-Embedded Ceramic Tablet as a Primary and Secondary Point-of-Use Water Purification Technology in Limpopo Province, S. Africa
Source: PLoS One. 2017 Jan 17;12(1):e0169502. doi: 10.1371/journal.pone.0169502 (PMC5240968; doi:10.1371/journal.pone.0169502)
Supplement: S13 Fig — Turbidity levels pre- and post-treatment among houses using ceramic water purification systems. Pre-treatment represented by influent samples and post-treatment samples represented by effluent samples. Data points represent average turbidity levels determined at weeks 37 and 52 combined. Standard error is used to calculate error bars. (PDF) [file pone.0169502.s013.pdf]

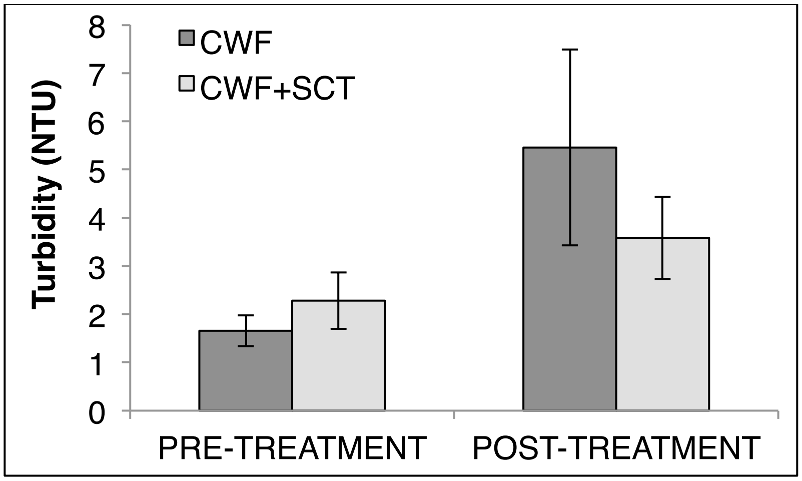

S13 Fig. Turbidity of ceramic-based technologies.
Turbidity levels pre- and post-treatment among houses using ceramic water purification systems. Pre-treatment represented by influent samples and post-treatment samples represented by effluent samples. Data points represent average turbidity levels determined at weeks 37 and 52 combined. Standard error is used to calculate error bars.
