# Supplementary material for: Evaluation of a Silver-Embedded Ceramic Tablet as a Primary and Secondary Point-of-Use Water Purification Technology in Limpopo Province, S. Africa
Source: PLoS One. 2017 Jan 17;12(1):e0169502. doi: 10.1371/journal.pone.0169502 (PMC5240968; doi:10.1371/journal.pone.0169502)
Supplement: S14 Fig — WTP was determined using binning method. (PDF) [file pone.0169502.s014.pdf]

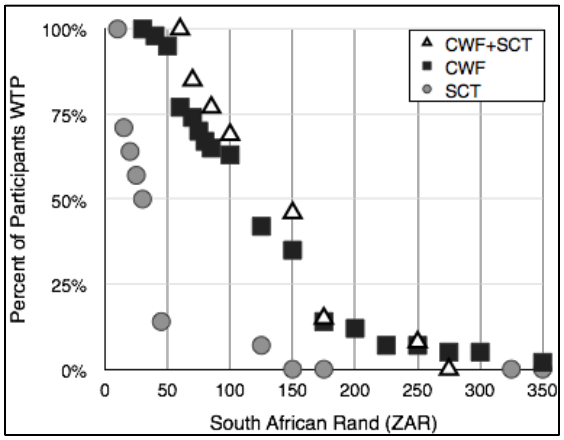

S14 Fig. Willingness-to-pay for each POU intervention (SCT, CWF and CWF+SCT) among 79 households in Limpopo Province, S. Africa.
WTP was determined using binning method.
