# Supplementary material for: Evaluation of a Silver-Embedded Ceramic Tablet as a Primary and Secondary Point-of-Use Water Purification Technology in Limpopo Province, S. Africa
Source: PLoS One. 2017 Jan 17;12(1):e0169502. doi: 10.1371/journal.pone.0169502 (PMC5240968; doi:10.1371/journal.pone.0169502)
Supplement: S15 Fig — (PDF) [file pone.0169502.s015.pdf]

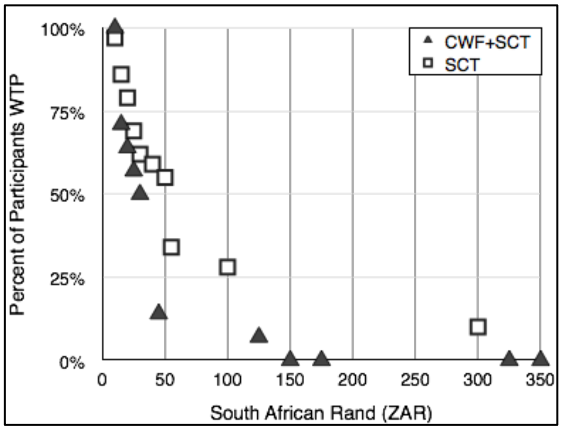


**S15 Fig. Comparison of willingness-to-pay for SCT among households using SCT as primary POU method (SCT) and as secondary POU method (CWF+SCT).**
