# Supplementary material for: Evaluation of a Silver-Embedded Ceramic Tablet as a Primary and Secondary Point-of-Use Water Purification Technology in Limpopo Province, S. Africa
Source: PLoS One. 2017 Jan 17;12(1):e0169502. doi: 10.1371/journal.pone.0169502 (PMC5240968; doi:10.1371/journal.pone.0169502)
Supplement: S1 Table — (PDF) [file pone.0169502.s016.pdf]

S1 Table. Number of Households sampled each week in CWF-only and CWF-SCT households

Week	Control Households	Intervention Households	Total Households
**1**	21	24	45
**2**	24	23	47
**3**	22	22	44
**4**	22	24	46
**5**	26	21	47
**37**	10	10	20
**52**	18	11	29
