# Supplementary material for: Evaluation of a Silver-Embedded Ceramic Tablet as a Primary and Secondary Point-of-Use Water Purification Technology in Limpopo Province, S. Africa
Source: PLoS One. 2017 Jan 17;12(1):e0169502. doi: 10.1371/journal.pone.0169502 (PMC5240968; doi:10.1371/journal.pone.0169502)
Supplement: S2 Table — (PDF) [file pone.0169502.s017.pdf]

S2 Table. Number of houses visited and samples collected each week among SCT-only households

Week	Number of houses visited	Number of samples
**1**	29	57
**2**	25	48
**3**	29	58
**4**	29	58
**5**	27	53
**37**	10	20
**52**	15	30
