# Supplementary material for: Evaluation of a Silver-Embedded Ceramic Tablet as a Primary and Secondary Point-of-Use Water Purification Technology in Limpopo Province, S. Africa
Source: PLoS One. 2017 Jan 17;12(1):e0169502. doi: 10.1371/journal.pone.0169502 (PMC5240968; doi:10.1371/journal.pone.0169502)
Supplement: S3 Table — (PDF) [file pone.0169502.s018.pdf]

S3 Table. Geometric mean of bacterial reduction among all three water purifications interventions each week.

	Total Coliform					*E. coli*		
	Week	Geometric mean	Standard Error	Number of participants		Geometric mean	Standard Error	Number of participants
Silver Embedded Tablet	1	60%	5%	47		98%	2%	11
	2	85%	3%	47		100%	0%	14
	3	80%	3%	50		92%	5%	11
	4	69%	4%	44		93%	4%	7
	5	72%	4%	38		100%	0%	9
	37	78%	9%	8		59%	26%	3
	52	81%	5%	9		64%	13%	7
Ceramic Water Filter Only	1	84%	5%	19		100%	0%	14
	2	98%	1%	19		100%	0%	11
	3	96%	3%	18		100%	0%	9
	4	85%	5%	18		100%	0%	10
	5	99%	1%	16		100%	0%	6
	37	99%	1%	10		100%	0%	7
	52	96%	3%	11		56%	16%	7
Ceramic Water Filter + Silver Embedded Tablet	1	95%	3%	21		100%	0%	13
	2	97%	2%	21		100%	0%	9
	3	91%	4%	16		100%	0%	8
	4	99%	0%	21		100%	0%	7
	5	89%	5%	15		99%	1%	7
	37	92%	6%	9		100%	0%	6
	52	93%	5%	7		100%	0%	3
