# Supplementary material for: Evaluation of a Silver-Embedded Ceramic Tablet as a Primary and Secondary Point-of-Use Water Purification Technology in Limpopo Province, S. Africa
Source: PLoS One. 2017 Jan 17;12(1):e0169502. doi: 10.1371/journal.pone.0169502 (PMC5240968; doi:10.1371/journal.pone.0169502)
Supplement: S4 Table — (PDF) [file pone.0169502.s019.pdf]

S4 Table. Baseline total coliform (TC) bacteria levels and water quality of samples among SCT households

	Week						
	1	2	3	4	5	37	52
**<u>Water Quality</u>**							
Number of Homes Showing Improvement in Water Quality	47 (82%)	41 (84%)	48 (86%)	41 (68%)	38 (69%)	8 (80%)	9 (60%)
Number of Homes Showing a Decline in Water Quality	9 (16%)	4 (8%)	8 (14%)	16 (27%)	14 (25%)	2 (20%)	6 (40%)
**<u>Number of Homes with Baseline TC levels:</u>**							
Less than 10 CFU/100mL	6 (11%)	10 (20%)	7 (13%)	6 (10%)	9 (16%)	1 (10%)	0 (0%)
From 10-49 CFU/100mL	6 (11%)	5 (10%)	9 (16%)	7 (12%)	9 (16%)	1 (10%)	7 (47%)
50-100 CFU/100mL	7 (12%)	8 (16%)	5 (9%)	8 (13%)	13 (24%)	4 (40%)	3 (20%)
More than 100 CFU/100mL	38 (67%)	26 (53%)	30 (54%)	39 (65%)	25 (45%)	4 (40%)	5 (33%)
**Total number of samples**	57	49	56	60	55	10	15
