# Supplementary material for: Evaluation of a Silver-Embedded Ceramic Tablet as a Primary and Secondary Point-of-Use Water Purification Technology in Limpopo Province, S. Africa
Source: PLoS One. 2017 Jan 17;12(1):e0169502. doi: 10.1371/journal.pone.0169502 (PMC5240968; doi:10.1371/journal.pone.0169502)
Supplement: S5 Table — (PDF) [file pone.0169502.s020.pdf]

S5 Table. Demographic Data

**Head of household**	
Median Age	44
Gender	
Male	8%
Female	92%
Average number of adults per household	2
Average number of children per household	3
**Education**	
Primary	22%
Secondary	62%
University	16%
**Monthly Income**	
Less than R250	7%
R250-1500	67%
Greater than R1500	26%
