# Supplementary material for: Evaluation of a Silver-Embedded Ceramic Tablet as a Primary and Secondary Point-of-Use Water Purification Technology in Limpopo Province, S. Africa
Source: PLoS One. 2017 Jan 17;12(1):e0169502. doi: 10.1371/journal.pone.0169502 (PMC5240968; doi:10.1371/journal.pone.0169502)
Supplement: S6 Table — (PDF) [file pone.0169502.s021.pdf]

S6 Table. Water practices

**Primary Water Source**	
Piped into yard	22%
Public tap/standpipe	55%
Borehole	18%
Surface water	5%
**Water storage vessel**	
Jerry can	35%
Plastic bucket (20-25 L)	34%
Water tank	1%
Plastic Drum (200 L)	22%
Plastic bottle	8%
**Where is water stored in home**	
Inside	95%
Outside	5%
Where inside?	
Kitchen	80%
**Cleaning method for water storage vessel**	
Boiled water	4%
Bleach	8%
Soap	57%
Nothing	32%
